# Disinformation From Within and Brandolini’s Law: A Call to Time-Consuming Action

**DOI:** 10.1016/j.focus.2025.100389

**Published:** 2025-06-30

**Authors:** Casey Scott Husser

**Affiliations:** Sanford Medical Center, University of South Dakota Sanford School of Medicine, Sioux Falls, South Dakota

## Abstract

**Figure:**
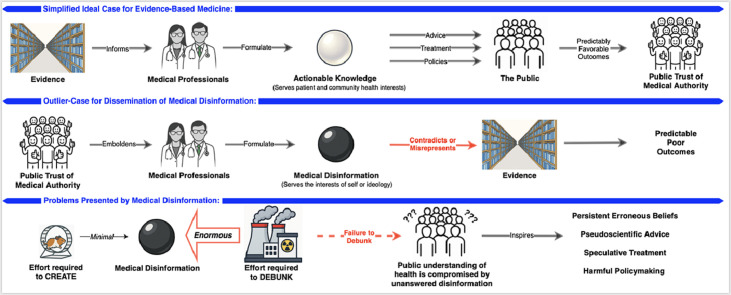

## INTRODUCTION

Scientific disinformation is a growing source of epistemic contamination that has taken root in the public’s perception of modern evidence-based medicine. When disinformation is spread by legitimate science, technology, engineering, and mathematics (STEM) professionals, the resultant damage is amplified owing to false-balance media coverage^1^ and gives the appearance of scientific disagreement where it may be trivial or nonexistent.^2^^,^^3^

A link to a nonpeer-reviewed article written by Vinay Prasad, MD, MPH, titled “A Simple Litmus Test for RFK Jr. ’s Ideas^1^” was shared to the author by a colleague in national security. The article is concerning, not only because it is replete with falsehoods and logical errors but because a professional with advanced academic credentials was left with the impression that its assertions were reasonable and factual. In my opinion, many of the article’s postulates conflict with HHS’s stated mission “to enhance the health and well-being of all Americans by fostering sound, sustained advances in the sciences underlying medicine, public health, and social services.”

This raised a troubling question: if even highly educated individuals can be persuaded to accept such medical disinformation, what hope remains for the broader, less scientifically sophisticated public? Unless the STEM community assumes a more active role in publicly debunking disinformation at every turn, the metastasis of erroneous beliefs will continue unabated.

The task of countering disinformation is paramount if facts and reason are to remain influential in an era when social media and memes increasingly shape public opinion on science. Although some scientists already engage with antiscience narratives on social platforms, there is a clear need for broader effort. Without compelling and intellectually robust responses from the scientific community, misleading information will continue to be absorbed as truth by larger segments of the public.

This paper offers evidence- or logic-based rebuttals to key claims presented in four of five sections of Prasad’s articulate but highly misleading article. Although these counterarguments strive to debunk factual and reasoning errors alike, the secondary intent of this paper is to inspire fellow scientists to contribute to public discourse and defend verifiable truth against the rising tide of popular, subjectivist disinformation, particularly when it originates from a member of our own community. A single expert’s appeal to their own authority is undermined whenever a consensus of experts can demonstrate in good faith why a claim of holding a novel and outlying scientific opinion might be nothing more than an eloquently delivered falsehood.

The unifying premise of Prasad’s article is that if a European country does something that RFK Jr. proposes as policy, then all should concede that said proposal is valid. This litmus test is arbitrary and fallacious, drawing conclusions that are disingenuous and unsupportable.

## METHODS IN BRIEF

Prasad’s essay was treated as the primary source document of study, containing 22 discrete, empirically testable assertions spread across five categories of his own choosing (the sixth and final category on nutrition was omitted for brevity). His claims were captured in order of appearance, ignoring purely rhetorical flourishes. For each claim, a search of the evidence was conducted through PubMed or Ovid, prioritizing results by high-quality systematic reviews/meta-analyses > randomized or prospective studies > authoritative consensus documents > retrospective reviews and, secondarily, by newer > older citations. Each claim was evaluated against the literature on four axes: overt factual accuracy, alignment with the preponderance of evidence, adequacy of contextual framing, and the presence and identification of logical fallacies. Rebuttals appear in the order presented by Prasad so that readers can crosswalk his claims to these rebuttals. This process, although tedious, provides a transparent, reproducible framework and guards against selective or arbitrary rebuttal.

### Raw Milk

RFK Jr. proposes removing restrictions for Americans to buy raw milk, and Prasad concurs despite ample evidence that the invention of pasteurization contributed to a 50% reduction in infant mortality,^4^ to say nothing of eliminating the suffering endured by the survivors of this historically common source of food poisoning.^2^^,^^5^, ^6^, ^7^, ^8^ Modern illness outbreaks are predominantly found among those who specifically seek out raw milk, as opposed to incidents of accidental contamination.^9^

Citing the popular cooking magazine *Bon Appétit* as his source, Prasad argues “many [raw milk] adherents believe the lack of processing makes the vitamins, minerals, enzymes, and fats easier for our bodies to absorb.”

Although adherents do hold beliefs, Prasad avoids explaining that none of those stated beliefs about raw milk are true, because pasteurized milk is just as nutritious as raw and avoids its dangers. Pasteurization can lead to a small decrease in certain vitamins, such as B1, B2, C, and folate, but can increase vitamin A levels.^10^ However, the overall impact on the nutritive value of milk is irrelevant because half of these vitamins are only present in low levels in raw milk to begin with,^11^^,^^12^ and fortification is neither difficult nor expensive.^13^ Pasteurization also does not significantly alter postprandial kinetics of proteins nor micronutrient content,^14^ making pasteurized milk as digestible as raw, if and only if one ignores the deleterious effects of food poisoning on digestion.^15^, ^16^, ^17^, ^18^ It is nonsensical to encourage raw milk consumption to obtain never-identified health benefits.

Prasad next argues that “Brown University economist Emily Oster calculates an annual risk of infection of 7 in 100,000 unpasteurized milk drinkers. That might be a risk some people choose to accept.”

This statistic checks out but sounds much less disturbing than the equally valid statistic that consumption of unpasteurized dairy products causes 840 times more illnesses and 45 times more hospitalizations than pasteurized dairy products.^3^ The fact that people choose to accept risk is not a valid argument in favor of safety. Most are innately poor at estimating risk-to-benefit ratios,^19^ which is the founding principle of lucrative industries built around risky yet psychologically rewarding activities such as gambling, recreational drug use, and motorcycling. As such, Prasad muses that the risks of raw milk are comparable with those of smoking and drinking alcohol, activities that no legitimate health professionals promote as beneficial to health.

Is the risk as trivial as Prasad suggests? Seven in 100,000 is a risk similar to that of developing other diseases such as infective endocarditis or myocarditis, the inciting risk factors for which are cautioned against rather than promoted.

The annual risk of developing infective endocarditis in the U.S. is 5.3–8.6 in 100,000.^20^^,^^21^ Disease burden would be higher if we encouraged people to participate in its risk factors, such as the avoidance of brushing one’s teeth^22^^,^^23^ or the reuse of needles when abusing intravenous drugs.^24^ Imagining that there are health benefits to poor oral hygiene or sharing needles is a parallel to Prasad’s argument in favor of increasing the population’s exposure to a predictable source of infection for the same reason. Raw milk is only consumed by 3.2% of the U.S. population but is responsible for over 96% of dairy-related illnesses.^5^ If health authorities encourage raw milk consumption, more people will consume it. Although the risk of infection per exposure will not necessarily change, the increase in total exposures will invariably lead to increases in foodborne illnesses, dragging down the average health of the population.

Seven in 100,000 is also equivalent to the yearly incidence of myocarditis from any cause in the general population (4–10 in 100,000).^25^^,^^26^ This is ironic because later in the article, Prasad singles out myocarditis as a condition so dire that large segments of the population should forgo vaccination against severe acute respiratory syndrome coronavirus 2 (SARS-CoV-2) to avoid it. Consider the following:

1. The risk of raw milk consumption is 7 in 100,000.
2. The risk of coronavirus disease 2019 (COVID-19) vaccine–mediated myocarditis is 0.38–2.1 cases per 100,000 doses.^27^^,^^28^
3. The risk of COVID-19 infection–mediated myocarditis is 276–4,000 cases per 100,000 infections.^29^

Prasad’s encourages citizens to take Risks 1 and 3, while openly fearing Risk 2, which has the lowest risk of all 3. The risk of disease from raw milk consumption is at least three times higher than the risk of vaccine-mediated myocarditis. The risk of more clinically severe infection-mediated myocarditis is up to 10,000 times higher than the risk of vaccine-induced myocarditis.^30^, ^31^, ^32^ Nonetheless, this exact disinformation routinely circulates as advice in social media^33^ and in politicized news commentary^34^ and is rarely scrutinized by scientists in public forums.

Prasad’s next argument is that because raw milk is legally available in some European countries, the U.S. should follow suit. This is not a scientific argument; it is the commission of *argumentum ad invidiam* or the envy fallacy. Cultivating desire for that which someone else has is not a logical argument in favor of lowered risk or elevated benefit because desire exists independently of risks and benefits. Furthermore, laws are less influenced by science than the unique history and culture of their originating country. This is obvious when observing foreign laws that contrast with one’s own cultural expectations:

1. Six European countries have fully socialized healthcare systems.^35^ This state of affairs has not converted the U.S. to conform.
2. Public utterance of profanity is punishable by 3–12 months in jail in the United Arab Emirates.^36^ This law would lead to unending incarceration for the author of this paper.
3. It is illegal to wear camouflage in 20 countries, including Ireland, where it is punishable by up to 3 months in prison.^37^
4. Japan is one of the kindest, most civilized nations on Earth, but if you are in possession of Adderall within their borders, even with a diagnosis of attention-deficit/hyperactivity disorder and a lawful prescription, you will be fined and imprisoned for up to 20 years.^38^ Obviously, you will serve that sentence unmedicated.
5. In most European nations (but in only 3 U.S. states), you can legally marry your first cousin.^39^ Why has Prasad neglected this cause, particularly when it is more widely adopted across Europe than even the legalization of raw milk?

A litmus comparison of how one culture observes or ignores the rules of dissimilar cultures is an illogical fact-finding strategy worthy of ridicule, given that science plays little to no role in crafting laws that are steeped in the prevailing attitudes of local customs.

Scientific knowledge is indispensable to creating public health policy. Cultural exceptions to observation of scientific principles made by any nation (including our own) are irrelevant to the veracity of the ignored facts. Therefore, consumption of raw milk is dangerous independently of the laws that govern it, because microbes are not subject to cultural norms. Conversely, Ireland’s stance against camouflage has no impact on the dress code at Bass Pro Shops in the U.S., because humans are heavily influenced by cultural norms.^40^

Prasad closes his argument on this topic by asserting that the unavailability of raw milk threatens liberty itself, stating “Americans are allowed to bungee jump, smoke cigarettes, and take part in all sorts of activities riskier than consuming raw milk. It is not the job of the state to eliminate all possible risks at the expense of pleasure.”

This ignores that advocates will feed their children raw milk. Children cannot legally smoke cigarettes, ride motorcycles, or drink alcohol. By law, adults have greater liberty to take risks than children, and Prasad makes no mention of what protections would exist for them. In fact, there are no such protections because adding raw milk to infant diets is actively promoted by fringe physicians^41^^,^^42^ through RFK Jr. ’s own Children’s Health Defense.^43^ Infants and young children are particularly vulnerable to pathogens found in raw milk and typically have worse outcomes than similarly exposed adults.^44^

The pleasure of raw milk consumption does not supersede a parent’s duty or the state’s responsibility to protect children from easily avoided life-threatening conditions. With sufficient exposure to cigarettes or heroin, children would come to find those pleasurable as well. Acquired pleasure is not a rational argument to allow children to smoke or partake of recreational opiates, nor should it be to feed them raw milk.

### The Measles, Mumps, and Rubella Vaccine

Prasad concedes that eliminating or reducing the routine pediatric use of measles, mumps, and rubella (MMR) vaccinations is not a good policy but only supports this conclusion because “all European countries recommend using MMR vaccines in children,” and because “No country I am aware of fears it leads to autism.”

This is the correct conclusion but omits that many high-powered studies independently conducted across the world and across tens of millions of children prove that there is no connection between autism and MMR vaccination^45^, ^46^, ^47^, ^48^, ^49^ nor their preservatives.^48^^,^^50^, ^51^, ^52^ Anyone with an MD or MPH (and he has both) should be able to cite evidence rather than checking their neighbor’s answers.

Prasad concludes with: “If RFK Jr. uses his perch as HHS secretary to discourage parents from getting their children inoculated with the MMR vaccine, severe negative repercussions could result, including measles outbreaks and childhood deaths.”

Although Prasad is more critical of RFK Jr. ’s position here than in any other section of his article, he fails to mention that RFK Jr. has already used his perch of celebrity to discourage parents from getting the MMR vaccine, contributing to the deaths of over 80 children in Samoa.^53^^,^^54^ The author struggles to understand why Prasad would simply hypothesize about a disaster that has already happened through the same person’s stance on the same vaccine. There are already devastating data available to cite without the need to imagine what might happen. RFK Jr. has tried to conceal his involvement in this tragedy,^55^ and Prasad is helping him in that ignoble effort by selective omission.

**COVID-19 policy.** Prasad endorses RFK Jr. ’s positions against mask mandates, against pediatric COVID-19 vaccination, and in denial that vaccines reduce transmission of disease.

A large body of evidence on masking, rigorously investigated by multiple, unrelated institutions, supports that it created measurable and significant decreases in the spread of disease,^56^^,^^57^ particularly indoors^58^^,^^59^ and in pediatric populations.^60^ It did not eliminate the risks of transmission, but even a bullet proof vest does not stop every kind of bullet.^61^ Criticism of this nature employs the Nirvana fallacy, where one argues in bad faith to suggest that anything short of perfect performance renders a solution completely useless.^62^ This remains as false in the case of seatbelts as it is in the use of masks. Vaccination was demonstrated to decrease susceptibility to infection and to reduce the infectiousness of breakthrough cases.^63^, ^64^, ^65^, ^66^ Falling short of sterilizing immunity does not mean that vaccination was not beneficial, as Prasad suggests.

Pediatric vaccination was robustly studied, and all evidence points to high efficacy in reducing the risk of infection and severe outcomes among the infected.^67^, ^68^, ^69^ The widely popularized higher risk of vaccine-induced myocarditis among males aged 12–17 years is frequently offered as evidence of harm without the key context that adolescent males are the single most susceptible demographic to viral myocarditis when infected,^70^ and they are over 10 times more likely to get myocarditis from infection than they are from vaccination.^71^^,^^72^ Viral myocarditis is much more severe in children and can lead to severe permanent cardiovascular complications and death.^32^^,^^73^ In contrast, most cases of vaccine-related myocarditis have a mild course with rapid resolution of symptoms and normalization of cardiac function.^74^

Prasad asserts: “Many European nations did not give Covid vaccines to kids, and that makes sense. Although the CDC was never willing to acknowledge this, children were at far lower risk from becoming infected than their elders.”

Although he is correct that children are less susceptible to infection and severe outcomes than adults,^75^^,^^76^ he again commits multiple factual and logical errors. The Centers for Disease Control and Prevention did not suppress this information, as evidenced by records as early as April of 2020.^77^^,^^78^

Prasad omits critical demographic context with respect to COVID-19 infection in children to claim that vaccination is unnecessary, which is false by virtue of spurious correlation. With respect to all-cause mortality, it is rare by more than an order of magnitude for children to die of anything in comparison to adults,^79^^,^^80^ and this is true even in the context of what children most frequently die of. Consider the following: 48,204 people in the U.S. died by firearm in 2022, and only 2,526 were children.^81^ Death by firearm has been the number 1 cause of child mortality since 2019, yet this is eclipsed by adult firearm mortality, which is only the 11^th^ most common cause of death among adults.^82^^,^^83^

Similarly, in 2020, 609,360 U.S. adults died of cancer, compared with only 1,600 children.^84^ This is not an artifact of differing population sizes because the mortality for cancer is approximately 110–298 per 100,000 adults^85^ and only 2.5 per 100,000 children.^86^

Pediatric COVID-19 mortality between August 2021 and August 2022 was among the 10 leading causes of childhood mortality in the U.S. It was 8th among all causes of death, 5th in disease-related mortality, and 1st in deaths caused by infectious or respiratory diseases compared with the rates in 2019.^87^ COVID-19 deaths represented 2% of all child deaths, yet Prasad concludes that “it makes sense” to do nothing to protect them from it. If that is the case, would it also make sense to remove age-related restrictions on the purchase and possession of firearms and to close and dismantle every pediatric cancer center?

The number of deaths among children is universally smaller than that among adults; Prasad’s assertion that lower mortality risks among children is a reason to cease protecting children from those risks is as flawed as it is morbid because it would demand that we cease protecting them from everything that they commonly die of.

Next, Prasad compares U.S. and Swedish COVID-19 mortality performance as a function of their respective mitigation strategies. Although the U.S. unquestionably did worse than Sweden, Prasad implies that U.S. mitigation measures caused this poorer performance. Many pandemic minimizers coveted Sweden’s limited restrictions policy, which kept most schools open and did not enforce masking.^88^ The impact on the health of Swedish nationals is clearer when comparing Sweden with its far more similar neighbor Norway, which had much stricter mitigation policies.

Sweden’s COVID-19–associated mortality rates during the first wave were 10 times higher than those in Norway, with Sweden reporting 2.9 deaths per 100,000 person-weeks compared with Norway’s 0.3.^89^ Sweden experienced greater overall mortality and healthcare system overload than its neighbor because of Sweden’s looser restrictions.^90^ Even the architect of Sweden’s policies openly expressed regrets over his country’s poorer performance.^91^

Ironically, Sweden still did better than the U.S. despite its lax mitigation possibly for one or more reasons. Swedes are generally more compliant with health recommendations than Americans, which made them more likely to follow public health advice.^92^^,^^93^ Obesity is a risk for poor COVID-19 outcomes,^94^ and the American population has nearly three times the obesity prevalence as Sweden.^95^^,^^96^ Sweden also had substantially more of their population vaccinated (by October 2021, over 75% of Swedes were vaccinated, compared with only 57% for Americans^97^^,^^98^). So paradoxically, Prasad argues against mitigation and vaccination on the basis of statistics that demonstrate superior performance in countries that had better mitigation compliance and vaccine coverage.

Prasad misquoted Pfizer’s profits as $100 billion in 2022 from vaccine sales alone. That was their total revenue for the year. Vaccine revenue only accounted for $38 billion.^99^ Vaccines were sold for $20–$30 per dose depending on the market—not as cheap as aspirin but not an overly expensive public health measure that saved an estimated 14–20 million lives the same year.^100^

Prasad endorses RFK Jr. ’s policy to revoke the National Childhood Vaccine Injury Act (NCVIA), which provides manufacturers indemnification from prosecution for real or perceived negative side effects.^101^ Vaccines have always had a low margin of profit for manufacturers^102^ and an often negative one for healthcare providers,^103^^,^^104^ all while providing massive benefits to personal and public health.^105^, ^106^, ^107^ Frivolous lawsuits in the 1970’s and 1980’s threatened vaccine supply owing to the imbalance of high costs and low financial returns.^108^ The NCVIA was a bipartisan effort signed into law by Ronald Reagan in 1986 to ensure an adequate supply of vaccines and stabilize vaccine costs and to establish a mechanism for compensating individuals who are injured by vaccines. The latter was accomplished by creating the National Vaccine Injury Compensation Program, a federal no-fault system funded by excise taxes on vaccines to compensate individuals or families of individuals injured by vaccines, without the use of the substantially more expensive and less evidence-based methodology of tort litigation.^109^

Overturning the NCVIA will lead to the exact issues that its creation sought to avoid: reduced vaccine access and weakened public health outcomes as vaccine-preventable illnesses dramatically increase rates of disability and death, particularly among children. This change would lead to significant negative effects on both national and global levels because many countries rely on the U.S. for vaccine policy, development, and production.^4^^,^^110^

### Fluoride

To support Prasad’s argument that RFK Jr. ’s fluoride policies are reasonable, he cites Tweets written by RFK Jr. This is despite the wide availability of scientific studies on the matter. This error poisons his conclusions with bias and circular reasoning because using RFK Jr’s written opinions to support RFK Jr’s political positions is a tragically compromised logic.

Prasad also cites an article in *The Economist* that covers RFK Jr. ’s proposed policy on water fluoridation. He correctly points out that there are risks to excessive consumption of fluoride, while carefully avoiding that similar risks exist for excessive consumption of nearly anything: ibuprofen,^111^ fat soluble vitamins such as vitamin A,^112^ and even water.^113^

Paracelsus’ fundamental principle of toxicology is that the dose makes the poison. The isolated existence of research-worthy toxicity is not a valid argument against any substance that is also beneficial or even mandatory for life itself. Prasad cherry picks the negative (albeit valid) points made against fluoridation, but the article clearly acknowledges that these correlations have not been demonstrated to be causative,^5^ and he avoids any discussion of the preventative health benefits of fluoridation.

Water fluoridation leads to a low-cost 25% reduction in the prevalence of dental caries among children and adults regardless of SES.^114^, ^115^, ^116^ Prasad ignores entirely the reasons for water fluoridation as if the prevention of dental caries is nothing more than a cosmetic goal. Untreated dental caries leads to serious systemic conditions such as infective endocarditis,^22^ intracerebral hemorrhage,^117^ ischemic stroke,^118^ adverse pregnancy outcomes such as macrosomia,^119^ and coronary artery disease.^120^ Water fluoridation indirectly mitigates the risk of these serious conditions through the prevention of tooth decay, highlighting its broader public health benefits beyond oral health.

Although there is evidence that suggests that abnormally high doses of fluoride pose a risk of small reductions in the intelligence quotients of children,^121^ particularly prenatally,^122^ the existence of this effect at normal concentrations found in municipal drinking water has not been convincing.^123^, ^124^, ^125^ Further research is warranted but appears unlikely to establish causation at standard municipal levels. Prasad’s concludes that “Germany, Norway, and Sweden don’t put fluoride in water [so] it is not crazy to think fluoride is unnecessary.”

As previously established, comparisons such as these are fallacious. Many laws that Germany, Norway, and Sweden collectively observe or compel of their citizens run contrary to those of the U.S., including the following:

1. public nudity (prohibited in the U.S. except in rare carve-outs);
2. allemansrätten: the right to roam, camp, and gather berries on private land (violates U.S. trespassing laws);
3. legal penalties for Holocaust denial (protected by the First Amendment);
4. government-mandated paternity leave (nonexistent in the U.S.); and
5. universal health care (nonexistent in the U.S.).

If Prasad seeks to argue on the grounds of bringing our laws and regulations more closely in line with those of our European neighbors, why has he failed to take on these other dissimilarities?

### Hepatitis B Vaccine

Prasad’s litmus test concludes that we should not vaccinate infants against hepatitis B virus (HBV) because several European countries do not. On the surface, this seems reasonable given that the endemicity of HBV is about as low in the U.S. as it is in the countries that choose targeted vaccination of the infants of high-risk pregnancies.^126^, ^127^, ^128^

Newborn vaccination against HBV intends to prevent perinatal (vertical) transmission of HBV, which leads to chronic infection 90% of the time^129^ and severe long-term complications such as cirrhosis, liver failure, cancer, and death.^130^ Vaccination confers long-term immunity 85%–95% of the time,^131^ which can protect children from future exposures to an otherwise incurable and often fatal disease for as long as 15 years without need of boosters.^132^

The context^133^ for why the U.S. chooses to implement immediate vaccination and Europeans do not is left out in Prasad’s article. Although disease prevalence are comparable across countries, access to and compliance with prenatal care are not. This leaves pregnant Americans far more likely to unwittingly transmit HBV to their newborn child.

The European countries that do not follow U.S. neonatal hepatitis guidelines (Denmark, Finland, Iceland, Norway, Sweden, and the United Kingdom) all have integrated and universally accessible healthcare systems. These countries generally achieve near-complete participation in early prenatal care,^133^ which allows ample time for screening and risk stratification. In the U.S., only 77.1% of women initiate prenatal care in the first trimester, with 15.0% of that total receiving inadequate prenatal care.^134^ It is for this reason that the U.S. universally vaccinates infants and, as such, has seen a 99% drop in cases of acute HBV among children,^135^ a 22% lower risk of all-cause mortality, and a 24% lower risk of cancer-related mortality than among unvaccinated individuals.^6^

Prasad speculates that “we need better evidence” on the safety and effectiveness of HBV vaccines, implying that they might be more harmful than beneficial. This is absurd, given the abundance of existing studies of safety and ongoing epidemiologic surveillance. There has never been a report of death in the U.S. that can be causally linked to vaccination against HBV.^136^^,^^137^ The same is true of studies conducted in China,^138^ Taiwan,^139^ Japan,^140^ and Italy.^141^ The availability of robust safety data on HBV vaccines is reassuring, particularly given the agreement in findings between countries with relationships that border on adversarial.

Prasad concludes this section with a proposal to use cluster RCTs of the entire childhood vaccine schedule to “allow researchers to account for additives or combined side effects,” implying that there would be no risks in performing this type of research on the population. This is not only hazardous but illegal for lack of informed consent if imposed by the edict of the director of the HHS.^142^

The contention of those who oppose vaccination against HBV is that vaccination carries risks that might equal or exceed its benefit. Prasad indirectly communicates this in stating that “doctors who say “all vaccines are safe and effective” are usually idiots.^7^” As established earlier, the efficacy of vaccination against HBV is very high, and the risk of severe adverse events are < 1 in a million^143^^,^^144^ in conferring protection against an infectious disease that is a leading cause of hepatocellular carcinoma^145^ and kills 1 in 100 of the infected.^146^ This is a false equivalence given that the risk of death from infection is 10,000 times greater than the risk of vaccination, and it calls to mind an analogy to skydiving, as follows:

The use of a parachute can independently cause injury and death. Line entanglements can cause severe injuries, and hard openings have led to lethal deceleration injuries in air that transect aortas and cervical spines alike.^147^ Using a parachute has risks, yet there are no RCTs for the safety and effectiveness of parachutes for high-altitude jumps,^148^ because doing so would ignore the reality that falling to the earth at terminal velocity (the control group) is by far the greatest risk in most (but not quite all) skydiving accidents.

Routine childhood vaccinations are no different than parachutes. They are safe and effective, and randomly enrolling children into a control group without consent by simply changing public policy will objectively expose our children to numerous preventable harms and deaths.^149^, ^150^, ^151^, ^152^ Controlled trials are only ethical to perform when there is no risk involved in giving human subjects a placebo or in the absence of known effective treatment.^153^ To suggest otherwise is to revisit the atrocity of the Tuskegee experiments on a national scale,^154^ where under the guise of providing health care, the natural histories of diseases that are already well understood are studied, while withholding safe and effective treatments in the form of vaccines from children.

## CONCLUSIONS

In 2013, an Italian programmer named Alberto Brandolini pointed out that the effort required to disprove disinformation is an order of magnitude greater than the effort to create it.^155^ Although the author had heard of the so-called Bullshit Asymmetry Principle before, he had never felt its sting until taking on this task. The author deferred on deconstructing the Additives in Food section because as demanded by both Brandolini’s Law and the finite nature of the universe, there is insufficient time and space to do so. The fact that Prasad unironically cited Food Babe as an expert should speak for itself. His article is only 2,300-word long, and the author’s best efforts to succinctly address only 80% of its disinformation is over 9,000. Although the author did not attain a full order of magnitude, no journal would publish a 23,000-word essay detailing the creative liberties that a professor of epidemiology and biostatistics would take with statistics and evidence in peer-reviewed literature.

Science and rationality are under attack, and they are on the defensive.^156^^,^^157^ There is a relentless onslaught of fallacious thinking and enticingly believable fairy tales about the natural world that have found solid purchase among the population. Over half of all Americans believe that genetically modified organism foods are unsafe, and that number has been larger on every survey since 2016^158^ despite ample evidence of safety.^159^^,^^160^ Over 40% of Americans believe that humans coexisted with dinosaurs.^161^ A total of 10% of U.S. residents doubt that humans have been to the moon,^162^ which is comparable with the number of Americans who believe that vaccines cause autism.^163^ This is our audience. If we as adherents to verifiable truth are silent, we risk ceding public discourse to those who favor sensationalism over substance, paving the way for anti-intellectualism to shape public policy.

When legitimate health scientists spread disinformation, public impact is more substantial and long lasting because field-appropriate professionals rightly appear more credible and trustworthy than outsiders.^164^^,^^165^ Andrew Wakefield is an archetype for this phenomenon. His fraudulent study falsely linking MMR vaccination to autism^166^ still fuels antivaccine sentiment decades after its 1998 publication.^167^^,^^168^ More recently, Judy Mikovits’ retracted 2009 study linking xenotropic murine leukemia virus–related virus to chronic fatigue syndrome^169^ cultivated notoriety in antiscience circles that later afforded her an opportunity to disseminate conspiracy theories about the safety of SARS-CoV-2 vaccines,^170^ which contributed to persistent vaccine hesitancy in the midst of a pandemic.^171^

Even benign health misinformation posited by professionals can linger for well over a half century, as evidenced by Robert Ho Man Kwok’s letter to the *New England Journal of Medicine* in 1968, in which he speculated that monosodium glutamate commonly used in Chinese cuisine might be the cause of numerous maladies.^172^ Even though double-blind placebo-controlled trials have failed to demonstrate a consistent and reproducible link between monosodium-glutamate consumption and adverse reactions, including IgE-mediated allergies,^173^ it remains a commonly reported allergy by patients.^174^

In 2016, Phil Williamson called for scientists to take a greater role in challenging disinformation.^175^ His message is critical in a world that is dependent upon science and technology and that is increasingly populated by citizens who lack even a rudimentary understanding of science or technology. Although you will never convince your direct adversary, you will sway part of the audience who lurks in silent observance, and those are the communication victories that science desperately needs to protect the public from dangerous ignorance.^176^ When the source of disinformation is someone who projects scientific authority, it is imperative that we firmly and thoughtfully correct the record, because fringe voices are given disproportionate attention in a culture that romanticizes outliers, both within^177^^,^^178^ and outside^179^ of academia.

The meek shall not inherit the Earth through quiet rationalism. The irrational are louder and far more successful at raising an audience through the spectacle of conflict or the illusion of oversimplified common sense. Reason must push back. If scientists hope to passively prevail because of a greater familiarity with verifiable truths, reason will lose to populism. There is an adage spuriously attributed to the Greek general Thucydides but actually coined by 19^th^ century biographer William Butler^180^ stating that “The nation that will insist on drawing a broad line of demarcation between the fighting man and the thinking man is liable to find its fighting done by fools and its thinking done by cowards.”

Modern use of this quote underscores the importance of integrating intellectual capability and the courage to take decisive action. The author contends that science benefits when scholars choose to take an active role in public discourse with the same dedication to scientific evidence and robust logic that is employed in each of our respective disciplines. You cannot be a scientist without refined intellectual skills, but your finest scientific efforts will go nowhere without the courage to defend them against forces that undermine facts in the name of political expedience or ideological compliance. Although this work is challenging, it is essential that we engage with the public to ensure a future founded on sound reason and verifiable truth.

### Limitations

Although unavoidably lengthy, this is not an encyclopedic survey of every argument Prasad has ever cited, nor does this manuscript exhaust the vaster depths of literature on the topics discussed. Inevitably, this leaves some assertions unaddressed—decisions made to keep the manuscript concise rather than to imply that those areas lack merit. The commentary also reflects the perspectives and disciplinary training of its author, a practicing physician and former biomedical informaticist. Alternative framings—economic, sociologic, patient advocacy—could yield different emphases. Although every effort was taken to cite the strongest, most recent evidence, the landscape of biomedical research is dynamic; conclusions should be revisited as new high-quality data emerge. The author encourages readers to view this work as a springboard for broader analyses and skepticism of the distortion of science for personal or political gain among STEM professionals rather than as an adjudication of the individuals who often gain notoriety from it.

## Footnotes

1 A Simple Litmus Test for RFK Jr. 's Ideas Accessed 2024/12/12.

2 A, Whitlock, L, Nichols M, Conrad, et al. Short Communication: Multistate Outbreak of Listeria Monocytogenes Infections Retrospectively Linked to Unpasteurized Milk Using Whole-Genome Sequencing. M Nichols, A Conrad, L Whitlock et al. Short communication: multistate outbreak of Listeria monocytogenes infections retrospectively linked to unpasteurized milk using whole-genome sequencing. *J Dairy Sci*. 2020;103(1):176–178. https://doi.org/10.3168/jds.2019-16703.

3 Costard S, Espejo L, Groenendaal H, Zagmutt FJ. Outbreak-related disease burden associated with consumption of unpasteurized Cow’s milk and cheese, United States, 2009–2014. *Emerg Infect Dis. Infect Dis*. 2017;23(6):957–964. doi:10.3201/eid2306.151603. https://doi.org/10.3201/eid2306.151603.Emerging.

4 United States vaccine research: A Delicate Fabric of Public and Private Collaboration. National Vaccine Advisory Committee. United States vaccine research: a delicate fabric of public and private collaboration. National Vaccine Advisory Committee. *Pediatrics*. 1997;100(6):1015–1020. https://doi.org/10.1542/peds.100.6.1015.

5 The Economist explains. *Should America Ban Fluoride in Its Drinking Water?* The Economist; 2024 (updated 2025/3/11).

6 He, Guo and Li. The Impact of Hepatitis B Vaccination in the United States, 1999–2018.

7 Cl A. Simple litmus test for RFK Jr. 's Ideas Accessed 2024/12/12.
